# Co-option of pre-existing vascular beds in adipose tissue controls tumor growth rates and angiogenesis

**DOI:** 10.18632/oncotarget.9436

**Published:** 2016-05-18

**Authors:** Sharon Lim, Kayoko Hosaka, Masaki Nakamura, Yihai Cao

**Affiliations:** ^1^ Department of Microbiology, Tumor and Cell Biology, Karolinska Institute, 171 77 Stockholm, Sweden; ^2^ Department of Medical and Health Sciences, Linköping University, 581 83 Linköping, Sweden; ^3^ Affiliated WuXi No 2 Hospital of Nanjing Medical University, Wuxi 214 002, China; ^4^ Department of Cardiovascular Sciences, University of Leicester and NIHR Leicester Cardiovascular Biomedical Research Unit, Glenfield Hospital, Leicester, LE3 9QP, UK

**Keywords:** adipose tissue, angiogenesis, tumor growth, vasculature, microenvironment

## Abstract

Many types of cancer develop in close association with highly vascularized adipose tissues. However, the role of adipose pre-existing vascular beds on tumor growth and angiogenesis is unknown. Here we report that pre-existing microvascular density in tissues where tumors originate is a crucial determinant for tumor growth and neovascularization. In three independent tumor types including breast cancer, melanoma, and fibrosarcoma, inoculation of tumor cells in the subcutaneous tissue, white adipose tissue (WAT), and brown adipose tissue (BAT) resulted in markedly differential tumor growth rates and angiogenesis, which were in concordance with the degree of pre-existing vascularization in these tissues. Relative to subcutaneous tumors, WAT and BAT tumors grew at accelerated rates along with improved neovascularization, blood perfusion, and decreased hypoxia. Tumor cells implanted in adipose tissues contained leaky microvessel with poor perivascular cell coverage. Thus, adipose vasculature predetermines the tumor microenvironment that eventually supports tumor growth.

## INTRODUCTION

Microvasculature distributed in various tissues exhibit marked differences in their density, structure, architecture, and functions. Among all tissues, adipose tissues, especially brown adipose tissue (BAT) is probably the most vascularized tissue in the body [1–4]. Each adipocyte is engulfed with vascular capillaries that form a high density of vascular network to nourish the adipocytes. Endothelial cells and perivascular cells such as pericytes are the two primary cell types that have direct interactions with adipocytes [2]. Additionally, endothelium of adipose vasculature contains fenestrae to mediate exchanges of hormones and other large molecules with the surrounding and remote tissues [5, 6]. Adipose tissue is probably the largest endocrine organ in the body and adipocytes produce various adipokines, hormones, growth factors, and cytokines to regulate its local microenvironment and the distal macroenvironment [4, 7–13]. Adipocytes constantly experience expansion and shrinkage according to their metabolic functions. To cope with alterations of adipocytes, the adipose vasculature exhibits marked plasticity during adipose tissue expansion and shrinkage [14, 15].

While white adipose tissue (WAT) stores excessive energy, BAT commits to energy expenditure by generating heat [16]. Under certain circumstances such as cold exposure, WAT, especially subcutaneous WAT, undergoes a browning transition and contributes to non-shivering thermogenesis [17–20]. This phenotypic and metabolic transition augments angiogenesis, which supplies fuel for thermogenesis [3, 17]. It is now known that endothelial cells produce various growth factors and cytokines to regulate adipocyte functions [4]. Additionally, vessel wall cells including endothelial cells and pericytes serve as a stem cell reservoir for supplying preadipocytes and adipocytes [21]. Adipocytes also produce various angiogenic factors including VEGF and angiopoietin to induce angiogenesis [2, 4, 17, 22, 23].

Tumor growth is dependent on angiogenesis that is stimulated by various tumor-derived angiogenic factors [24–27]. Inhibition of tumor angiogenesis is an effective approach for treatment of human cancers [24]. Tumors originating from different tissue microenvironments are exposed to different vascular beds. The pre-existing vasculature in various tissues would provide an uneven opportunity for tumor angiogenesis, growth, invasion, and possibly drug responses [28, 29]. In this present study, we provide compelling evidence to demonstrate that tumors implanted in highly vascularized WAT and BAT tissues exhibit accelerated tumor angiogenesis and growth. Thus, pre-existing vessels stimulate tumor development, angiogenesis and growth rate. The clinical implication of our findings may suggest that tumors originating from adipose-associated tissues have a growth advantage and may respond differently to anti-cancer drugs.

## RESULTS

### Differential breast cancer growth in subcutaneous tissue, WAT and BAT

**Figure 1:**
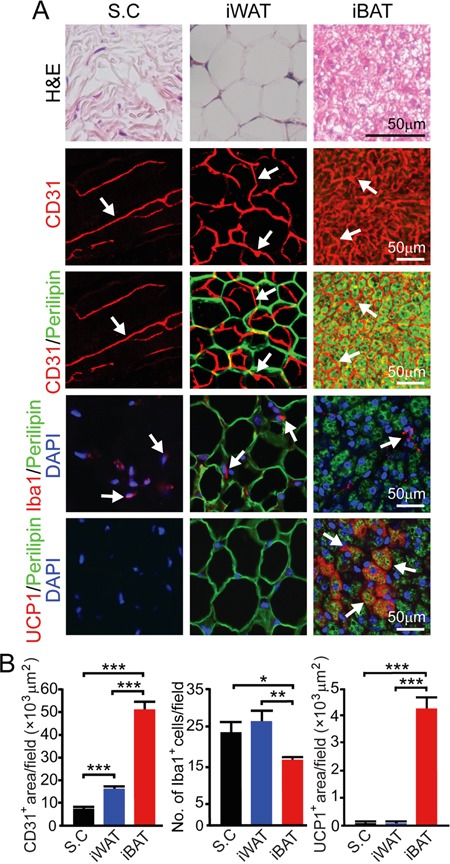
Vascularity in subcutaneous tissue, iWAT and iBAT **A.** H&E, CD31, CD31 plus perilipin, Iba1 plus perilipin and UCP1 plus perilipin staining of subcutaneous tissue (S.C), iWAT and iBAT of adult healthy C57BL/6 mice (n = 8 mice/group). Representative micrographs are shown. Arrows point to microvessel, UCP1, and Iba1 positive signals in their corresponding panels. Bar = 50 μm. **B.** Quantification of CD31^+^ microvessel density (n = 8 random fields/group), number of Iba1^+^ (n = 6-8 random fields/group), and UCP1^+^ signals (n = 6 random fields/group). *p<0.05; **p<0.01; ***p<0.001. Data were presented as mean determinants ± S.E.M.

To investigate the variation of pre-existing vascular beds in different tissues in modulating tumor growth, we chose subcutaneous tissue, WAT and BAT for our study. Immunohistochemical staining of these tissues show that inguinal WAT (iWAT) and interscapular BAT (iBAT) contained substantially higher density of microvessel than the subcutaneous tissue (*P* < 0.001) (Figure 1A). Microvessel density of iBAT was approximately 4-fold higher than that in iWAT (*P* < 0.001) (Figure 1B). Under the experimental temperature condition (22-23°C), a substantial number of iBAT adipocytes expressed UCP1, which is a key mitochondrial protein responsible for non-shivering thermogenesis (Figure 1A and 1B). It appeared that iBAT contained fewer inflammatory macrophages relative to subcutaneous and iWAT tissues (*P* < 0.01) (Figure 1A and 1B).

**Figure 2:**
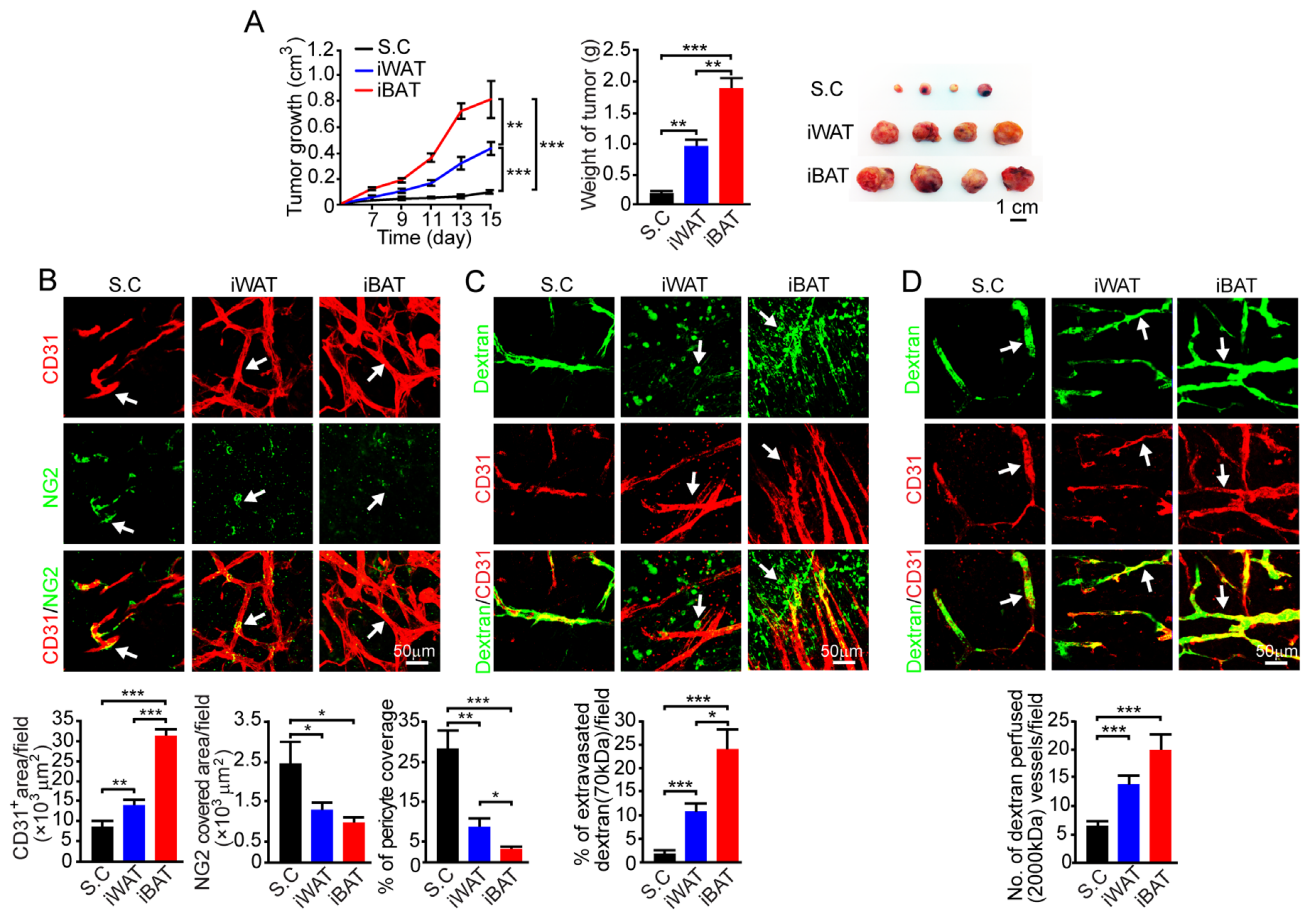
Growth rate, angiogenesis, permeability, and blood perfusion of EO771 mouse mammary tumors **A.** Approximately 0.5 × 10^6^ tumor cells were implanted in subcutaneous region of the dorsal back, iWAT and iBAT of each adult mouse. Tumor growth rates were measured as volume and weighed (n = 8 mice/group). Representative tumors are shown. **B.** CD31^+^ microvessel density and morphology, NG2^+^ pericytes associated area and % NG2^+^ pericyte coverage microvessel of tumors implanted in subcutaneous tissue, iWAT and iBAT. Arrows indicate tumor microvessel, pericytes, and pericyte coverage in their corresponding panels. Bar = 50 μm. Quantification of CD31^+^ microvessel density, NG2^+^ pericytes associated area, and % of NG2^+^ pericyte coverage (n = 8 random fields/group). **C.** Extravasation of rhodamine-labeled 70-kDa dextran in subcutaneous, iWAT and iBAT implanted tumors. Arrows point to the extravasated dextran signals. Bar = 50 μm. Quantification of the extravasated dextran positive signals (n = 6 random fields/group). **D.** Blood perfusion of 2000-kDa dextran in subcutaneous, iWAT and iBAT implanted tumors. Arrows point to the dextran-perfused vessels. Bar = 50 μm. Quantification of the dextran-perfused positive signals (n = 6 random fields/group). *p<0.05; **p<0.01; ***p<0.001. Data were presented as mean determinants ± S.E.M.

For studying tumor growth, we chose an EO771 mouse breast cancer model because breast cancer development and progression are tightly associated with adipose depots. Implantation of 0.5 × 10^6^ EO771 cells in the dorsal subcutaneous region of syngeneic C57BL/6 mice resulted in a relative slow growth rate. At 15 days after tumor implantation, the average tumor size was less than 0.1 cm^3^ (Figure 2A). Notably, implantation of the same number of EO771 cells in iWAT resulted in a marked accelerated tumor growth rate relative to subcutaneous implantation (*P* < 0.001) (Figure 2A). Implantation of tumor cells in iBAT led to an exceptionally fast growth, which was nearly 10-fold greater than subcutaneous tumors. While tumors implanted in subcutaneous tissue had a delayed onset of tumor takeoff, iWAT and iBAT showed accelerated tumor takeoff. Particularly, implantation of tumor cells in iBAT resulted in almost immediate tumor growth without the delayed tumor takeoff period. These findings demonstrate that tumors implanted in the highly vascularized adipose tissues exhibit accelerated growth rates and tumors in iBAT show an exceptional growth advantage.

### Alterations in the tumor microenvironment

We investigated angiogenesis in tumors grown in different tissues and discovered that the degree of tumor vascularization reconciled with the degree of pre-existing microvessel density. In iWAT, a nearly 2-fold increase of microvessel density was observed as compared to that of subcutaneous tumors (*P* < 0.01) (Figure 2B). In iBAT tumors, an exceptional high vessel density was detected. Additionally, it appeared that microvessel displayed a dilated and disorganized phenotype in iBAT tumors, which also lacked pericyte coverage. In concordance with the disorganization phenotype and lack of pericyte coverage, tumor vessels in iWAT and iBAT were highly leaky of 70-kDa dextran (*P* < 0.01) (Figure 2C). Consistent with augmented tumor angiogenesis, blood perfusion was also proportionally increased in iWAT and iBAT (*P* < 0.001) (Figure 2D).

**Figure 3:**
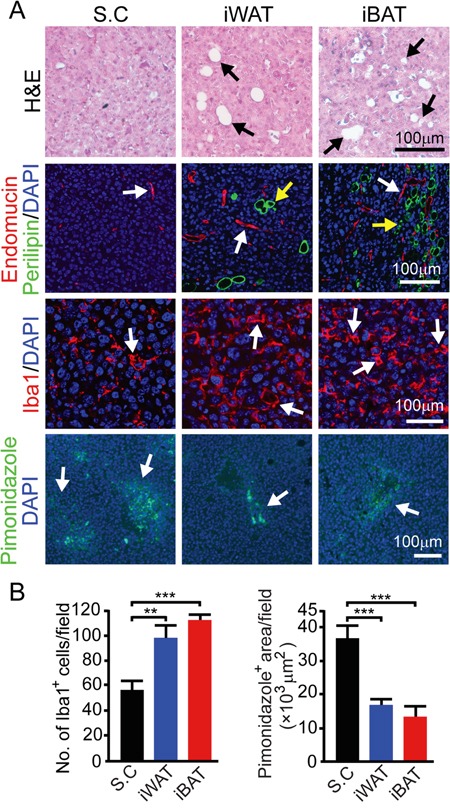
Microvessel, inflammation, and tissue hypoxia of EO771 mouse mammary tumors implanted in subcutaneous, iWAT and iBAT tissues **A.** Representative micrographs of H&E, endomucin plus perilipin, Iba1, and pimonidazole staining. Arrows point to adipocytes, microvessel, Iba1 and pimonidazole positive signals. Bar = 100 μm. **B.** Quantification of number of Iba1^+^ (n = 8 random fields/group), and pimonidazole^+^ signals (n = 6 random fields/group). **p<0.01; ***p<0.001. Data were presented as mean determinants ± S.E.M.

We next investigated the microenvironment of tumors grown in different tissues. In both iWAT- and iBAT-implanted tumors, we found intimate interactions between tumor cells and adipocytes. Substantial numbers of adipocytes were present in tumor tissues and they showed positivity for perilipin (Figure 3A). Another interesting finding is that tumors in iWAT (*P* < 0.01) and iBAT (*P* < 0.001) contained markedly higher numbers of tumor-associated macrophages (TAMs) (Figure 3A and 3B). Consistent with increased blood perfusion, tumor tissues implanted in iWAT and iBAT showed decreased in tissue hypoxia (*P* < 0.001) (Figure 3A and 3B).

### Melanoma growth in subcutaneous, iWAT and iBAT tissues

**Figure 4:**
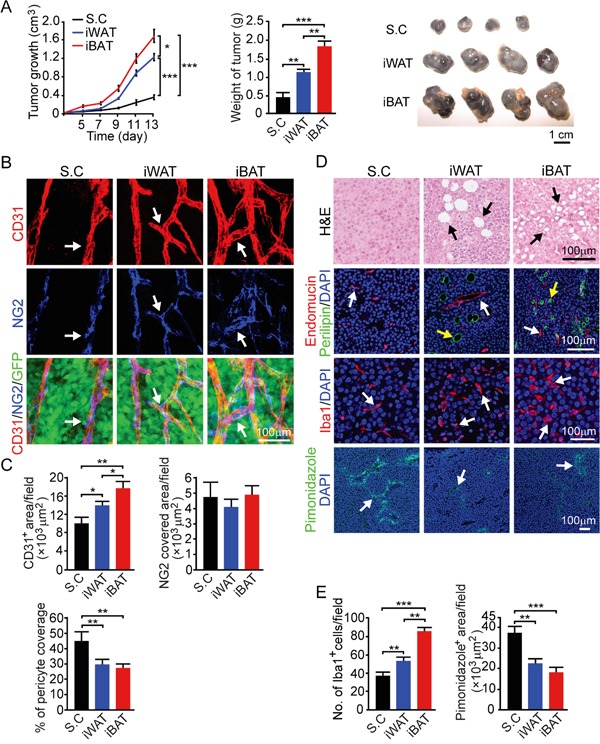
Growth rate, angiogenesis, microvessel, inflammation and tissue hypoxia of B16-F10 mouse melanoma tumors implanted in subcutaneous, iWAT and iBAT tissues **A.** Approximately 0.5 × 10^6^ tumor cells were implanted in subcutaneous region of the dorsal back, iWAT and iBAT of each adult mouse. Tumor growth rates were measured as volume and weighed (n = 8 mice/group). Representative tumors are shown. **B.** CD31^+^ microvessel density and morphology, NG2^+^ pericytes associated area and % NG2^+^ pericyte coverage microvessel of tumors implanted in subcutaneous tissue, iWAT and iBAT. Arrows indicate tumor microvessel, pericytes, and pericyte coverage in their corresponding panels. Bar = 100 μm. **C.** Quantification of CD31^+^ microvessel density, NG2^+^ pericytes associated area, and % of NG2^+^ pericyte coverage (n = 8 random fields/group). **D.** Representative micrographs of H&E, endomucin plus perilipin, Iba1, and pimonidazole staining. Arrows point to adipocytes, microvessel, Iba1 and pimonidazole positive signals. Bar = 100 μm. **E.** Quantification of number of Iba1^+^ (n = 8 random fields/group), and pimonidazole^+^ signals (n = 6 random fields/group). *p<0.05; **p<0.01; ***p<0.001. Data were presented as mean determinants ± S.E.M.

We chose a melanoma model to further validate our findings from the breast cancer model since melanoma, especially invasive melanoma, exhibits vertical growth which is often in associated with the adipose tissue. In principle, the melanoma model reproduced the findings from the breast cancer model (Figure 2). Similar to the breast cancer model, implantation of mouse B16-F10 melanoma in iWAT and iBAT syngeneic C57BL/6 mice resulted in marked accelerated growth rates compared to subcutaneous tumors (*P* < 0.001) (Figure 4A). Again, tumors implanted in the iBAT showed advantages of tumor takeoff and growth, which were significantly greater than tumors implanted in the iWAT. Consistetly, iWAT and iBAT tumors were highly vascularized with reduced pericyte coverage compare to subcutaneous tumors (Figure 4B and 4C). Tumors-implanted in iWAT and iBAT also showed decreased in hypoxia, higher numbers of Iba1 positive cells (*P* < 0.01 in iWAT and *P* < 0.001 in iBAT), and infiltration of adipocytes (Figure 4D and 4E). These findings provide additionally supportive evidence that tumors in adipose tissues possess quick takeoff and accelerated growth rates.

### Fibrosarcoma growth in subcutaneous, iWAT and iBAT tissues

**Figure 5:**
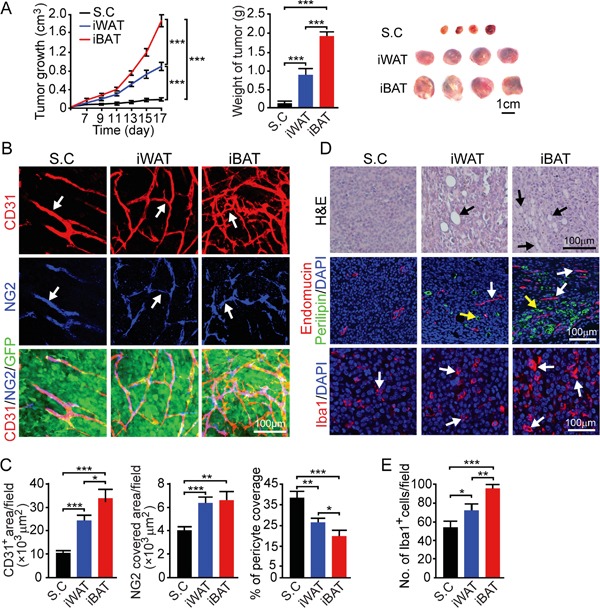
Growth rate, angiogenesis, microvessel and inflammation of T241 mouse fibrosarcoma tumors implanted in subcutaneous, iWAT and iBAT tissues **A.** Approximately 0.5 × 10^6^ tumor cells were implanted in subcutaneous region of the dorsal back, iWAT and iBAT of each adult mouse. Tumor growth rates were measured as volume and weighed (n = 8 mice/group). Representative tumors are shown. **B.** CD31^+^ microvevessel density and morphology, NG2^+^ pericytes associated area and % NG2^+^ pericyte coverage microvessel of tumors implanted in subcutaneous tissue, iWAT and iBAT. Arrows indicate tumor microvessel, pericytes, and pericyte coverage in their corresponding panels. Bar = 100 μm. **C.** Quantification of CD31^+^ microvessel density, NG2^+^ pericytes associated area, and % of NG2^+^ pericyte coverage (n = 8 random fields/group). **D.** Representative micrographs of H&E, endomucin plus perilipin and Iba1 staining. Arrows point to adipocytes, microvessel and Iba1 positive signals. Bar = 100 μm. **E.** Quantification of number of Iba1^+^ (n = 8 random fields/group). *p<0.05; **p<0.01; ***p<0.001. Data were presented as mean determinants ± S.E.M.

We chose a third tumor model, mouse fibrosarcoma T241 to further validate our findings. Consistent with the results from breast and melanoma models, similar observations were seen in the fibrosarcoma T241 model: higher pre-existing vascular density in iWAT and iBAT supports faster tumor takeoff as compared to tumors implanted subcutaneously (Figure 5A). Tumor growth rates were significantly faster in tumors implanted in the iWAT and iBAT (*P* < 0.001) (Figure 5A). The microvessel density of tumors implanted in iWAT and iBAT was significantly higher than that of subcutaneous tumors (*P* < 0.001) (Figure 5B and 5C). Similarly, pericyte coverage in iWAT and iBAT were reduced as compared to the subcutaneously implanted tumors (Figure 5B and 5C). Fibrosarcoma tumors implanted in the iWAT and iBAT also demonstrated increased infiltration of adipocytes and Iba1 positive cells (Figure 5D and 5E). These results further validate our findings that higher pre-existing vascular density in the adipose tissues initiates tumor takeoff and supporting tumor growth.

## DISCUSSION

Our present work provides novel insights on the pre-existing vascular niches in different tissues in supporting tumor takeoff and growth. Among other dissimilarities, vascular beds in various tissues are different in numbers, structures and functions. Despite this knowledge, the role of pre-existing vasculature in the initial tumor formation and subsequent growth remains elusive. Our data show that implantation of tumor cells in highly vascularized iWAT and iBAT tissues results in an accelerated tumor takeoff. In support of this view, some of the most common human cancers including breast cancer, prostate cancer, colorectal cancer, and pancreatic cancer are all originating from the adipose environment. High incidences of these cancers could reflect the fact that they are likely newly formed cancers that escaped dormancy. It is also known that an anatomical barrier made of fibrous septae often exists surrounding the tumor mass in the adipose environment. Perhaps, the fibrous septae serves as the protective structure for tumor cell invasion and accelerated growth. In fact, tumors which have the fibrous septae are often pathologically defined as benign tumors even though malignant cells may have similar genetic alterations.

Another interesting aspect of adipose associated cancers is that malignant cells might be able to switch their metabolic programs toward a lipogenic pathway rather than solely glucose-dependent pathways. It is known that the lipogenic pathway provides excessive energy for tumor cell proliferation and migration, leading to an accelerated growth rates and invasiveness [37]. Therefore, the adipose microenvironment not only provides high density of microvessel that support tumor formation and growth, but could also reprogram of tumor cells metabolism to support invasion.

Genetic mutations of oncogenes and tumor suppressor genes occur frequently in many other non-adipose-associated tissues as well. However, these tumors can remain dormant for years without growth and may not even progress into clinically detectably mass. An outstanding example is the thyroid cancer, where in situ microscopic carcinomas are present in virtually all thyroid glands of autopsied individuals ages between 50 to 70 [38]. These cancer patients can live for years and even the rest of their lives without developing clinically manifested cancer disease. Assuming that a dormant thyroid tumor is present in an adipose tissue microenvironment, this tiny tumor would have grown into angiogenic tumor within a relatively short time. This interesting possibility warrants further investigation. Another interesting aspect of adipose tissues in facilitating tumor growth is the unique metabolic profiles of adipocytes and immune response. It is likely that certain types of cancer cells are able to utilize lipid for generation of sufficient energy for their proliferation and mobility, leading to invasion and metastasis. Also, the abundant number of inflammatory cells in the adipose environment could facilitate cancer invasion and metastasis.

Our findings could be further expanded to metastatic cancer growing in highly vascularized mesentery environment where the adipose tissue is a major component. It is known that metastatic tumors grow in the mesentery at an accelerated rate and contain a high density of microvessel [39]. The high density of microvessel in adipose tissues would provide vascular niches for tumor cell co-option. In fact, the initial formation of a microscopic primary tumor or a metastatic niche is attained by co-option of the pre-existing vascular network in a given tissue [40–42]. Even a microscopic tumor with only a few hundreds of cells is dependent on nutrient and oxygen supply from the pre-existing vasculature by free diffusion. However, further expansion of a tumor size beyond a few cell layers requires intratumoral neovascularization [40]. It is likely that a high number of pre-existing microvessel would result in an enhanced angiogenic response, which would accelerate tumor growth. In this regard, the pre-existing vasculature stimulates subsequent tumor growth.

Treatment of adipose tissue-associated cancers with antiangiogenic drugs may result in differential responses due to the vascular plasticity in the adipose tissue. Some cancer types such as pancreatic cancer growing in the adipose tissue are completely resistant to antiangiogenic therapy. Perhaps, antiangiogenic drugs such as bevacizumab do not target the proangiogenic factors in the adipose tissue. Another example is the breast cancer patients who did not show overall survival benefits from anti-VEGF-based antiangiogenic drugs, resulting in the withdrawal of previously approved bevacizumab for treatment [43]. It warrants further investigation and clinical validation to demonstrate if this is a general trend of drug response for adipose-related cancers.

## MATERIALS AND METHODS

### Animals

Female C57BL/6 mice at age between 6-8-week-old were obtained from the breeding facility of the Department of Microbiology, Tumor and Cell Biology at Karolinska Institute, Stockholm, Sweden. All animal studies were approved by the North Stockholm Experimental Animal Ethical Committee.

### Cell culture

Monolayers of EO771 tumor cells were cultured in Roswell Park Memorial Institute (RPMI) 1640 medium (HyClone; catalog no. SH30027.01), supplemented with 10% (vol/vol) heat-inactivated fetal bovine serum (FBS) (HyClone; catalog no. SH30160.03) and penicillin-streptomycin (100 U/mL) (HyClone; catalog no. SV30010). Monolayers of B16-F10 and fibrosarcoma T241 tumor cells were cultured in Dulbecco's modified Eagle's medium (DMEM) (HyClone; catalog no. SH30243.01), supplemented with 10% (vol/vol) heat-inactivated FBS and penicillin-streptomycin (100 U/mL). All cell lines were purchased from the ATCC. All cell lines were not authenticated after purchase or transferred from other laboratories but were routinely tested negative for mycoplasma by using the Mycoplasma Detection Kit (Lonza).

### Mouse tumor models

Cultured tumor cells at the concentration of 0.5 × 10^6^ cells in 30 μL of phosphate-buffered saline (PBS) were subcutaneously (s.c.) injected into the dorsal back along the midline of each mouse in the Dorsal S.C group (n = 8 mice). To visualize adipose depots, small subcutaneous incisions were created in regions of mouse iWAT and iBAT (n = 8 mice per group). Approximate 0.5 × 10^6^ tumor cells in 30 μL PBS were injected into the iWAT depot or the iBAT depot of each mouse, followed by closure of incisions with sterile surgical suture (Ethicon; VICRYL V422 4-0). For pain relief, operated mice received twice daily for 2 days s.c. injection of Temgesic (RB Pharmaceuticals) (0.1 mg/kg). Tumor sizes were measured every other day with a caliper and tumor volumes were calculated according to a standard formula: length × width^2^ × 0.52). At the end of each experiment, mice were sacrificed by cervical dislocation. Tumors were dissected and tumor tissues were immediately fixed at 4°C overnight with 4% paraformaldehyde (PFA), followed by washing with PBS prior to whole-mount staining.

### Vascular permeability, perfusion and hypoxia probe labeling

The hypoxia probe pimonidazole hydrochloride (Hypoxyprobe; catalog no. HP2-1000) at a dose of 60 mg/kg was intravenously (i.v.) injected into each mouse that was sacrificed 30 minutes later. Tumor tissues were dissected and immediately fixed in 4% PFA. Lysinated rhodamine-fixable dextran (LRD) at a molecular weight of 70- or 2000-kDa (Invitrogen, catalog no. D1818 and D7139) was intravenously injected into the tail vein of each tumor-bearing mouse when the tumor size reached 0.8 cm^3^. At 5 minutes after injection with 2000-kDa dextran and 15 minutes after injection with 70-kDa dextran, mice were sacrificed, and tumor tissues were immediately fixed in 4% PFA overnight and further analyzed by whole-mount staining.

### Whole-mount staining

Tissue whole-mount staining was performed as described previously [30–32]. Briefly, fresh tumor tissues fixed at 4°C overnight with 4% PFA were washed with PBS. Tumor tissues were cut into small and thin pieces using a scalpel blade, followed by digestion with proteinase K (20 mM in Tris buffer, pH 7.5) for 5 minutes. Tissues were permeabilized with 100% methanol for 30 minutes and blocked a PBS-based blocking buffer containing 3% milk and 0.3% Triton X-100 (Sigma-Aldrich; catalog no. X100). Tissue samples were double immunostained at 4°C overnight with a rat anti-mouse CD31 antibody (BD Pharmingen; catalog no. 553370; 1:200) and a rabbit anti-mouse NG2 antibody (Millipore; catalog no. MAB5384; 1:200). Stained tumor tissues were further blocked with 3% milk, followed by incubation at room temperature for 2 hours with secondary antibodies including: a goat anti-rat Alexa 555 (Invitrogen; catalog no. A21434; 1:400); a goat anti-rat Cy5 (Millipore; catalog no. AP183S; 1:400); or a goat anti-rabbit Cy5 (Millipore; catalog no. AP132S; 1:400). Tissues were thoroughly washed with PBS at 4°C overnight before mounting in Vectashield mounting medium (Vector Laboratories; catalog no. H-1000). Samples were stored at −20°C in dark until images were captured using a confocal microscope (Nikon D-Eclipse C1, Nikon Corp.). Quantitative analyses were performed from the data of at least 6-8 different random fields using the Adobe Photoshop CS5 program.

### H&E staining

Paraffin-embedded tissues were sectioned at 5 μm in thickness and baked at 60°C for 1 hour. Tissue samples were deparaffinized using Tissue-Clear (Sakura; catalog no. 1466) and hydrated with decreasing concentration of ethanol. Samples were stained with hematoxylin (Sigma-Aldrich; catalog no. MHS16-500ML), rinsed with water for 10 minutes, and counterstained with Eosin Y solution (Sigma-Aldrich; catalog no. 318906) followed by dehydration and mounted with Pertex (HistoLab; catalog no. 00801). Stained tissues were examined using a light microscope (Nikon Eclipse TS100, Nikon Corp.).

### Immunohistochemistry

Paraffin-embedded tissues were sectioned at 5 μm in thickness and baked at 60°C for 1 hour. After deparaffinization and hydration, tissue antigens were retrieved by boiling for 15 minutes in an unmasking solution (Vector Laboratories; catalog no. H3300). Tissue samples were washed three times with PBS, followed by blocking with 4% a non-immune serum (Vector Laboratories; catalog no. S-1000), and were stained overnight at 4°C with primary antibodies including: a rat anti-mouse Endomucin (eBioscience; catalog no. 14-5851-85; 1:100); a rabbit anti-mouse UCP1 (Abcam; catalog no. ab 10983; 1:100); a rabbit anti-mouse Iba1 (WAKO; catalog no. 019-19741; 1:400); and a Guinea pig anti-mouse perilipin (Fitzgerald; catalog no. 20R-PP004; 1:400). Tissue samples were incubated at room temperature for 45 min with species-matching fluorescein-labeled secondary antibodies: a goat anti-rat Alexa 555 (Invitrogen; catalog no. A21434; 1:400), a donkey anti-rabbit Alexa 647 (Invitrogen; catalog no. A31573; 1:400), and a goat anti-guinea pig Alexa 649 (Jackson Immunogen; catalog no. 106-495-003; 1:400). For detection of tumor hypoxia, a FITC-conjugated mouse anti-pimonidazole monoclonal antibody (Hypoxyprobe; catalog no. clone 4.3.11.3; 1:400) was used. Tissue slides were mounted with Vectashield mounting medium containing DAPI for nuclear staining (Vector Laboratories; catalog no. H-1200) and stored at −20°C in dark until images were captured using a fluorescence microscope (Nikon digital sights, Nikon Corp.).

### Image analysis

CD31^+^ microvessel area per field, NG2^+^ covered pericyte area per field and pimonidazole^+^ area per field, were quantified using the Adobe Photoshop CS5 program [33–36]. The positive signals were detected as pixels using the color range tool. The pixel numbers displayed on the histogram window were recorded and converted to micrometer square per field using the Microsoft Excel program. The number of Iba1^+^ cells per field was counted manually using the count tool in the Adobe Photoshop program.

### Statistical analysis

Data represent means ± SEM. *P* values were determined by unpaired Student's *t* test. *P < 0.05 was considered significant.
